# A Comprehensive Review and Update on the Pathogenesis of Inflammatory Bowel Disease

**DOI:** 10.1155/2019/7247238

**Published:** 2019-12-01

**Authors:** Qingdong Guan

**Affiliations:** ^1^Department of Immunology, University of Manitoba, Manitoba, Canada; ^2^Department of Internal Medicine, University of Manitoba, Manitoba, Canada; ^3^Cellular Therapy Laboratory, Cancer Care Manitoba, Manitoba, Canada; ^4^Research Institute in Oncology and Hematology, Cancer Care Manitoba, Manitoba, Canada; ^5^Manitoba Centre for Advanced Cell and Tissue Therapy, Manitoba, Canada

## Abstract

Inflammatory bowel disease (IBD) is a chronic and life-threating inflammatory disease of gastroenteric tissue characterized by episodes of intestinal inflammation. The pathogenesis of IBD is complex. Recent studies have greatly improved our knowledge of the pathophysiology of IBD, leading to great advances in the treatment as well as diagnosis of IBD. In this review, we have systemically reviewed the pathogenesis of IBD and highlighted recent advances in host genetic factors, gut microbiota, and environmental factors and, especially, in abnormal innate and adaptive immune responses and their interactions, which may hold the keys to identify novel predictive or prognostic biomarkers and develop new therapies.

## 1. Introduction

Inflammatory bowel disease (IBD) is a chronic inflammatory disease of the gastrointestinal tract, which clinically contains Crohn's disease, ulcerative colitis, and other conditions [1]. The inflammation of the intestinal mucosa in IBD is characterized by episodes of abdominal pain, diarrhea, bloody stools, weight loss, and the influx of neutrophils and macrophages that produce cytokines, proteolytic enzymes, and free radicals that result in inflammation and ulceration [1, 2].

IBD is a lifelong disease occurring early in life in both males and females. The incidence and prevalence of IBD markedly increased over the second half of the 20th century, and since the beginning of the 21st century, IBD has been considered one of the most prevalent gastrointestinal diseases with accelerating incidence in newly industrialized countries [3–5]. The highest prevalence of IBD was reported in Europe (ulcerative colitis 505 per 100,000 persons in the southeast of Norway; Crohn's disease 322 per 100,000 persons in Hesse, Germany) and North America (ulcerative colitis 286.3 per 100,000 persons in Olmsted County, USA; Crohn's disease 318.5 per 100,000 persons in Nova Scotia, Canada) [5]. Since 1990, the incidence rate of IBD in Western countries was shown to be stable or started to drop, but the incidence rate in newly industrialized countries of Asia, Africa, and South America was increasing [5].

Crohn's disease usually involves the terminal ileum, cecum, perianal area, and colon, but it can affect any region of the intestine in a discontinuous pattern [6–8]. In contrast, ulcerative colitis involves the rectum and can affect part of the colon or the entire colon in a continuous pattern [6–8]. Crohn's disease exhibited histologically a thickened submucosa, transmural inflammation, fissuring ulceration, and granulomas, whereas the inflammation in ulcerative colitis is limited to the mucosa and submucosa with cryptitis and crypt abscesses [7–9].

Although the cause of IBD remains unknown, considerable progress has been made in recent years to unravel the pathogenesis of this disease. Studies have provided evidence that the pathogenesis of IBD is associated with genetic susceptibility of the host, intestinal microbiota, other environmental factors, and immunological abnormalities [10, 11].

## 2. Pathogenesis of IBD

### 2.1. Genetic Factors

Genome-wide association studies (GWAS), next generation sequencing studies, and other analysis have identified over 240 nonoverlapping genetic risk loci, of which around 30 genetic loci are shared between Crohn's disease and ulcerative colitis [12–14]. The analysis of the genes and genetic loci identified in IBD indicates that several pathways play important roles in maintaining intestinal homeostasis, such as epithelial barrier function, innate mucosal defense, immune regulation, cell migration, autophagy, adaptive immunity, and metabolic pathways associated with cellular homeostasis [8, 15–17]. The permeability of the epithelial barrier enables microbial incursion, which is recognized by the innate immune system, which then launches appropriate tolerogenic, inflammatory, and restitutive responses partially by secreting extracellular mediators that recruit other cells, including adaptive immune cells [8].

Nucleotide-binding oligomerization domain 2 (NOD2) is the first gene found to be associated with Crohn's disease, which is frequently mutated in patients with Crohn's disease, occurring in around one-third of the patients [18, 19]. For instance, Crohn's disease patients associated with 1007fs mutation in the NOD2 gene show a much more severe disease phenotype than other Crohn's disease patients, while R702W and G908R mutations lead to increase inflammatory cytokine responses [6]. NOD2, a member of the cytosolic Nod-like receptor (NLR) family based on their triggers and the signaling pathways that they control, is one of the two important and distinct detection systems to sense microbial invaders [6]. NLR proteins are found in the cytoplasmic compartment, and the other detection systems are membrane-bound receptors, termed toll-like receptors (TLRs). NOD2 can recognize the minimal bioactive fragment of peptidoglycan found in the cell wall of both Gram-negative and Gram-positive bacteria, called muramyl dipeptide (MDP) [6, 20, 21]. Thus, NOD2 is thought to be important as an intracellular sensor of bacterial components [6, 20, 21]. Upon binding to its ligand—MDP, a conformational change of NOD2 occurs that allows it to bind the caspase recruitment domain of the adaptor protein RIP2 [6, 20]. RIP2 then induces the polyubiquitination of nuclear factor kappa B (NF-*κ*B) essential modulator—I*κκγ*, which is the key scaffolding protein of NF-*κ*B [20]. It then activates NF-*κ*B, leading to secretion of some proinflammatory cytokines, such as IL-12. It can also activate the MAPK signaling pathway [6, 20].

NOD2 has also been implicated in the initiation of autophagy [8, 22]. Autophagy is a highly conserved recycling process involving the degradation of cytosolic contents and organelles, as well as resistance against infection and the removal of intracellular microbes [8, 22]. MDP stimulation can activate the autophagy process leading to confinement of intracellular bacteria within autophagosomes and subsequent control of infection [23]. Following bacterial recognition, NOD2 serve as molecular scaffolds for the nucleation of the autophagy machinery by interacting with ATG16L1 [23]. ATG16L1 is essential for all forms of autophagy. Interestingly, ATG16L1 polymorphisms are also linked to Crohn's disease like NOD2 [23]. The variant encoding the T300A substitution in *ATG16L1* increases the susceptibility of the protein ATG16L1 to caspase-3 cleavage and decreases its function [17, 24]. In patients with Crohn's disease who are homozygous for the T300A substitution in *ATG16L1*, they have abnormal TLR signaling and Paneth cell function [17]. Selective deletion of *ATG16L1* in T cells in mice results in spontaneous intestinal inflammation characterized by aberrant Th2 responses to dietary and microbiota antigens and decreasing Foxp3^+^ Treg cell number [25]. These impaired T cell responses contribute to the disruption of the mucosal barrier through breaking the tolerance to intestinal antigens and promoting the secretion of IgG and IgA against commensal microbiota [17, 25].

GWAS has identified numerous single-nucleotide polymorphisms (SNP) in *IL-23R*, with high association for Crohn's disease and ulcerative colitis [26, 27]. Of interest, Arg381Gln, an uncommon allele at a highly conserved amino acid polymorphism, confers a protective effect in patients with Crohn's disease or ulcerative colitis through modulating IL-23R recycling and cytokine production by macrophages [27, 28].

The majority of risk-associated loci are shared across populations, but some loci show heterogenicity between populations; for example, *NOD2* and *IL23R* variants are present in the majority of European patients, but not in East Asian ancestry patients [29]. Also, although many individuals carry IBD-associated risk loci, only a small population develops IBD. Therefore, additional environmental factors and alterations to the interactions between the gut microbiota and mucosal immune system are required for the development of IBD.

### 2.2. Gut Microbial Factors

IBD appears to result from abnormal host immune responses to the intestinal microbiota [30–32]. Intestinal microbiota is the major environmental driver of IBD. The gastrointestinal tract of the human body is colonized at birth by a vast range of microorganisms that numerically exceed host cells by around 10 times [32, 33]. The gut contains 1000-5000 different species, with 99% coming from Firmicutes, Bacteroidetes, Proteobacteria, and Actinobacteria [30]. These microorganisms contain around 100-fold as many genes present in the human genome [30, 32, 33]. The gut microbiota can be influenced by diet, probiotics, prebiotics, antibiotics, exogenous enzymes, fecal microbiota transplantation, and other environmental factors [31].

This gut microbiota is necessary for intestinal homeostasis and function, health, and disease [32–34]. Tolerance to gut microbiota must be maintained to benefit from their coexistence; on the contrary, colonization with specific pathogenic microbes might be detrimental to the host, leading to disease [33]. The coexistence with the microbiota can be beneficial to host metabolism and gastrointestinal development [32, 33]. In addition, the commensal microorganisms are required for the development and differentiation of the local and systemic immune system and nonimmune components [32, 33]. They can protect the host from enteric pathogenic infections via colonization resistance and via synthesis of factors promoting mutualism [32]. For example, induction of a transforming growth factor- (TGF-) *β*-rich environment by indigenous *Clostridium species* enhances regulatory T cell (Treg) numbers and function in the colon and the resistance to DSS-induced murine colitis [32, 35]. Therefore, the host has evolved numerous mechanisms to maintain the homeostasis.

Both commensal and pathogenic microorganisms determine the consequence of an infection. Microbes can be detected by recognition by pattern recognition receptors (PRRs, including TLRs, NLRs, C-type lectin receptors, and RIG-like receptors) of pathogen-associated molecular patterns (PAMPs), which are found in many species of microorganisms [31–33]. The recognition of PRRs activates the innate immune system, leading to the activation of NF-*κ*B and inflammasome, which stimulates the production of proinflammatory cytokines and chemokines, and this can also enhance tissue homeostasis and mucosal tolerance in the absence of barrier broken [33, 36]. The PAMPs are small molecular motifs conserved within many species of nonpathogenic and pathogenic microbes. Therefore, PRRs recognition is largely unable to distinguish between nonpathogenic and pathogenic microbes [33, 37]. It leads to the recognition of innate immunity to commensal microorganisms, having a crucial role in the maintenance of intestinal homeostasis, and is critical for the protection against gut injury and associated mortality [33, 37]. The imbalance of these interactions contributes to the development of intestinal inflammation.

In the complexity and multiplicity of the gut microbiota, our understanding on the roles of commensal and pathogenic microorganisms in establishing a healthy intestinal epithelial barrier and in disrupting the intestinal homeostasis has been greatly increased in the past decades [31, 36]. Animal studies have demonstrated that the intestinal microbiota play both proinflammatory and anti-inflammatory roles in the pathogenesis of IBD, and in most animal colitis models, the intestinal microbiota is indispensable for driving pathogenesis [36]. However, in human, it is difficult to demonstrate a definitive cause-effect relationship between intestinal microbiota and IBD [36]. Based on the studies in human and animal infection models, it is unlikely that a single infection causes or triggers the IBD in humans. But the intestinal microbiota clearly promotes the development of IBD [32, 33]. For instance, the presence of *Mycobacterium avium* subsp. *paratuberculosis* and adherent-invasive *Escherichia coli* is increased in Crohn's disease patients; the presence of *Clostridium difficile* is increased in both Crohn's disease and ulcerative colitis patients in relapse and remission states [38]. The increased mucosal bacterial counts and decreased anti-inflammatory commensal *Faecalibacterium prausnitizii* are also found in Crohn's disease patients [38].

In summary, microbial factors play important roles in the pathophysiology of IBD through impacting the immune systems in major ways and affecting host metabolism and gastrointestinal development [31–33, 36].

### 2.3. Environmental Factors

The important role of environmental factors in the pathogenesis of IBD is supported by recent studies on IBD epidemiology. The frequency of Crohn's disease has significantly increased in the more developed countries over the past 50 years, and the recognition of the disease corresponding with progressive industrialization in the less developed countries has also increased [39, 40].

Food intake is an important environmental factor that affects the development of IBD [41]. Studies have provided evidence that intake of fruit and vegetable has been associated with decreased risk of Crohn's disease [42]; intake of fast foods containing many fat and sugar-rich foods may exacerbate the development of Crohn's disease [41]. One study also shows that medium-chain fatty acids are more effective in accelerating intestinal inflammation than long-chain fatty acids [43]. In most of Western developed countries, sugar-rich foods have been recognized as one of the risk factors for Crohn's disease [41], and artificial food additives prevalent in Western diets may promote intestinal inflammation by interfering with barrier function in the gut [42].

Smoking is another example of a disease-specific modifier that seems to worsen Crohn's disease while being protective against ulcerative colitis [39, 40]. Smoking has been shown to affect cellular and humoral immune responses and to promote colonic mucus production [39, 41]. Nicotine, an essential content of cigarettes, has an inhibitory effect on Th2 cell function, but has no effect on Th1 cell function [39]. Evidence also suggests that smoking impairs autophagy, a process thought to be involved especially in Crohn's disease [44].

There are other environmental factors that influence the development of IBD, including but not limited to psychological stress, appendectomy, diet, and medications [45]. For example, appendectomy is an independent risk factor for developing Crohn's disease, while it is protective for ulcerative colitis [46]. Although many epidemiological studies already identified those environmental factors with disease evolution of IBD, it is still facing challenges to explore the mechanism studies of how environmental factors impact IBD disease progress [45]. One study shows that diet rich in animal protein promotes proinflammatory macrophage responses and exacerbates murine colitis [47].

### 2.4. Immunological Abnormalities

The immunological dysregulation in IBD is characterized by epithelial damage (abnormal mucus production, defective repair); expansion of inflammation driven by intestinal flora and a large number of cells infiltrating into the lamina propria including T cells, B cells, macrophages, dendritic cells (DCs), and neutrophils; and a failure of immune regulation to control the inflammatory response [3, 48, 49]. The activated lamina propria cells produce high levels of proinflammatory cytokines in the local tissue, including TNF, IL-1*β*, IFN-*γ*, and cytokines of the IL-23/Th17 pathway [3, 7, 48].

The intestinal immune system is divided into innate immunity and adaptive immunity. Innate immunity includes the barrier function of the intestinal mucosa, antibacterial proteins (complement, defensins, etc.), the acid PH value of stomach to limit microbial growth, innate immune cells (neutrophils, macrophages, DCs and natural killer T cells, etc.), and innate cytokines and molecules (IL-1, TNF, and defensins) [49]. Adaptive immunity is pathogen-specific and is usually initiated under the circumstances in which the innate immune responses cannot circumvent the stimulation of a pathogen [49]. After exposure to a pathogen, it usually takes several days to finally activate adaptive immune responses, including T and B cells [49]. The initiation of immune response to intestinal flora is tightly regulated, and this regulation determines the occurrence of immune tolerance or a defensive inflammatory response. Disturbance of the balance of these responses can cause IBD [7].

#### 2.4.1. Dysregulation of the Innate Immune System

*(1) Intestinal Epithelial Barrier*. The 400 mm^2^ single layer of intestinal epithelial cells (IEC) is the primary cellular barrier. It functions as a selective barrier to confine the entry of antigens to the mucosal immune system for the aim of inducing oral tolerance to commensal microorganisms or food antigens and for the aim of host defense against pathogens [18, 50]. Therefore, the IEC play important roles in the gut in an immunological context through providing the antigen-sampling machinery, expressing PPRs (e.g., TLR and NLR) and involving the establishment of the tolerogenic environment in the intestine and controlling of the immune system in the gut-associated lymphoid tissue (GALT) [51]. The tight junctions between epithelial cells allow for the selective penetration of nutrients, fluids, and microorganisms. Normal gastrointestinal permeability relies on the intact epithelium, surface mucus, and peristalsis and the production of host protective factors [52].

Epithelial integrity is disturbed in IBD patients, and mice that have deficient epithelial barrier functions develop colitis [18]. The absorptive cells in colonic crypts expressing the proton channel OTOP2 and the satiety peptide uroguanylin, which can sense pH, is dysregulated in IBD [53]. In IBD, intestinal epithelial goblet cells are positionally remodeled and coincided with the downregulation of WFDC2, which is an antiprotease molecule with the ability to preserve the integrity of tight junctions between epithelial cells and prevents invasion by bacteria and mucosal inflammation [53]. One GWAS study identifies 3 susceptibility loci related to the epithelial barrier function in ulcerative colitis patients: *HNF4A* regulating the expression of cell junctions; *CDH1* encoding E-cadherin, a main component of adherent junctions; and *LAMB1* encoding laminin beta 1 subunit, expressed in the intestinal basement membrane [48]. Proinflammatory cytokines, secreted during intestinal inflammation such as TNF or IFN-*γ*, can increase the epithelial permeability by regulating tight junctions and promoting apoptosis [18]. IFN-*γ* increases paracellular permeability and induces endocytosis of tight junction transmembrane proteins [54]. Increased permeability to macromolecules has been found in IBD patients [55]. The high apoptotic rate of epithelial cells also leads to diminished epithelial barrier function observed in IBD. Studies have shown that apoptotic rate is increased in mildly to moderately inflamed colon of Crohn's disease and ulcerative colitis [18]. Also, apoptosis allows the loss of ions and water and the entry of small antigens [56]. IL-13, a key effector Th2 cytokine in ulcerative colitis, also shows the ability to impair epithelial barrier function by affecting epithelial apoptosis, tight junctions, and reconstitution velocity [57]. The reduced velocity of restitution can play a role in the response of an epithelial layer to naturally occurring or pathogen-induced small lesions [57].

The intestinal epithelium is also responsible for electrolyte transport. Disrupted electrolyte transport may lead to diarrhea [18]. Around 50% of Crohn's disease patients and almost 100% of ulcerative colitis patients have diarrhea as a symptom. The deficiencies of electrolyte transport in IBD contain hyporesponsiveness of electrogenic anion secretion, reduced synthesis of epithelial sodium channels, reduced NaCl absorption, and alteration of electrochemical gradient [18].

The intestinal epithelium may be improved, protected, and repaired by growth factors and cytokines [58]. These growth factors and cytokines play vital roles in the regulation of cell proliferation, differentiation, angiogenesis, inflammation, intestinal defense mechanisms, and intestinal wound repairs [18]. Currently, at least 30 different peptide growth factors have been shown to be involved in the maintenance of intestinal mucosal integrity, including epidermal growth factor, the TGF-*β* family, the insulin-like growth factor family, the fibroblast growth factor family, and the colony-stimulating factor family [58]. Of these factors, epidermal growth factor, insulin-like growth factor family, fibroblast growth factor family, and colony-stimulating factor family appear promising in the treatment of IBD and are being evaluated in clinical trials [18].

*(2) Dendritic Cells*. DCs are hemopoietic bone marrow progenitor-derived leukocytes, which are widely distributed throughout the body in small numbers [59, 60]. Although DCs were first described by *Paul Langerhans* in the late nineteenth century, their role as a central coordinator was not established until 1973 by *Ralph Steinman* et al. [61, 62]. DCs are professional antigen-presenting cells (APCs) specialized in antigen capture, process, and presentation to T cells. DCs are considered to be the most potent APCs that orchestrate innate and adaptive immune responses [63].

DCs are found throughout the gut, including the lamina propria, isolated lymphoid follicles, Peyer's patches, and mesenteric lymph nodes (MLNs) [60]. DCs have been documented both in the maintenance of immune tolerance to the commensal microorganisms and food antigens and in the initiation of host defense against pathogens [64, 65]. In the intestine, DC subtypes have been characterized into conventional DCs and plasmacytoid DCs, similar to those in other peripheral lymphoid organs [51, 59]. Conventional DCs are further divided into the following: CD11b^+^CD8*α*^−^ DCs in the subepithelial dome, preferentially secreting IL-10 and inducing Th2 cells; CD11b^−^CD8*α*^+^ in the interfollicular regions; and CD11b^−^CD8*α*^−^ subsets in both areas, preferentially secreting IL-12 and inducing Th1 cells [51, 59]. Plasmacytoid DCs are specialized in the production of type I interferons [65]. In the steady-state lamina propria, two major DC subsets have been characterized based on the reciprocal expression of CD103 and CX_3_CR1 [51, 59].

DCs are present in an immature state with high phagocytic ability localized in peripheral tissues and in discrete regions of organized secondary lymphoid organs [59]. Immature DCs constitutively acquire foreign and self-antigens from the intestinal lumen through the following: (1) microfold (M) cells which transcytose antigens from the lumen to the mucosa [59]; (2) CX_3_CR1^+^ DCs extending dendrites between IEC and into the intestinal lumen to directly capture antigens and present them to CD4^+^ T cells, which differentiate into effector T cells and secret proinflammatory cytokines [59, 60, 64]; (3) direct sample antigens as a result of breaches in the epithelial integrity as seen in intestinal inflammation [59, 64]; (4) mechanisms mediated by the fetal Fc receptor [59, 64]; and (5) lamina propria CD103^+^ CX_3_CR^−^ DCs receiving conditioning from epithelial cells and serving as the inducer of Treg cells [59, 60, 64].

After capturing antigens, immature DCs migrate from the Peyer's patch and lamina propria to the draining MLN, where they present the antigens to naïve T cells [51]. During the migration, DCs gradually become mature with the expression of costimulatory molecules. In addition, the lamina propria DCs constitutively transport antigens from apoptotic IEC or commensal microorganisms to the draining MLN to interact with T and B cells to initiate tolerogenic responses [51]. In particular, CD103^+^ DCs isolated either from the lamina propria or from the MLN promote the development of Foxp3^+^ Treg, which rely on retinoic acid and TGF-*β* [60]. Also, DCs conditioned in the presence of IEC-secreted thymic stromal lymphopoietin (TSLP) are less capable of secreting IL-12 and promoting Th2 responses [51].

In the presence of pathogens, the migration of DCs to the MLN increases. Activated DCs trigger a protective immune response including activating effector cells and determining which CD4^+^ T helper cells (e.g., Th1, Th2, or Th17) will predominate [64].

In patients with IBD, DCs are attracted by the upregulated chemokines such as CCL20 or addressins such as mucosal vascular addressin cell adhesion molecule-1 and accumulate at inflammatory sites. Correlating with large amounts of DCs accumulation in the intestine, plasmacytoid DCs and myeloid DCs are downregulated in the peripheral blood of patients with active IBD [60, 64]. In the lesions of Crohn's disease, the numbers of CD83^+^ DC and DC-specific ICAM-3 grabbing nonintegrin (DC-SIGN)^+^ populations are significantly increased, while IL-12 and IL-18 are only detected in DC-SIGN^+^ DC and not in CD83^+^ DC [66]. DCs from MLN of patients with Crohn's disease preferentially induce the Th1 response [67]. Three types of DC are identified in the MLN of Crohn's disease and ulcerative colitis patients, including mature DCs, myeloid DCs, and plasmacytoid DCs [67]. Myeloid DCs from MLN of patients with Crohn's disease produce high levels of IL-23 and low levels of IL-10 [67]. While plasmacytoid DCs are shown to infiltrate intestinal mucosa of IBD patients, one recent murine study shows that plasmacytoid DCs are largely dispensable in the pathogenesis of intestinal inflammation during IBD [68].

Besides reacting inappropriately to captured antigens, intestinal DCs might also receive inappropriate signals from IEC during intestinal inflammation [60]. IEC isolated from about 70% of patients with Crohn's disease do not express TSLP mRNA and cannot control the DC-mediated proinflammatory response, leading to upregulated production of IL-12 by DCs, which then polarizes Th1 responses [69]. NOD2 expression on DCs may also play a critical role in their responses to microbes, because DCs derived from NOD-2 deficient Crohn's disease patients have an impaired ability to induce IL-17 production upon MDP challenge [70].

Evidence from animal models also demonstrates the role of DCs in the chronic intestinal inflammation. Large amounts of activated DCs accumulate in the lamina propria and MLN in murine models of colitis [60, 64]. In the CD45RB^hi^ CD4^+^ T cell transfer model of colitis, large amounts of CD11c^+^ DCs expressing activation marker OX40 ligand (OX40L) are found in the MLN, and transferred T cells create aggregates with CD11c^+^ DCs in the lamina propria. Blocking OX40-OX40L interaction ameliorates colitis [71, 72]. Analysis of the DC phenotype in murine colitis has shown that colonic lamina propria mature DCs express higher levels of costimulatory molecules (CD40, CD80, and CD86) and increase productions of IL-12p40 and IL-23p19 upon CD40 ligation [73]. IL-12p40 and IL-23p19 form IL-23, which is important for the stabilization of Th17 cell activation. When DCs are selectively ablated in mice before developing dextran sodium sulfate- (DSS-) induced colitis, the colon inflammation is exacerbated when compared with that of untreated mice [59].

Taken together, these data indicate that DCs play an important role in the pathogenesis of IBD through influencing the tolerance to the commensal microflora and dietary antigens and affecting immune responses [64].

*(3) Myeloid-Derived Suppressor Cells (MDSC)*. MDSC are a heterogeneous population of cells that expand during cancer and other pathogenic conditions and have a remarkable ability to suppress various T cell responses and promote Treg expansion [74, 75]. MDSC suppress immunity by perturbing both innate and adaptive immune responses through secreted soluble mediators and induction of Treg cell expansion [74, 76]. MDSC contribute to the failure of immune therapy in patients with cancer and have been considered a therapeutic target for the treatment of cancer [74, 77, 78]. The expansion and functional importance of MDSC in noncancer pathogenic conditions have been recently recognized. MDSC numbers are imbalanced and they act as a downregulation mechanism of immune responses in many diseases, such as autoimmune diseases [79], transplantation [80, 81], and asthma [82, 83, 84].

The roles of MDSC in IBD have been demonstrated in IBD patients and animal models. MDSC are increased in the peripheral blood of IBD patients [85, 86]; CD14^+^HLA-DR^-/lo^ monocytic MDSC have the ability to suppress T cell proliferation [85], while CD33^+^CD15^+^ granulocytic MDSC fail to suppress T cell response but instead enhance T cell proliferation [86]. We and others report that MDSC are increased in TNBS- or DSS-induced murine colitis, and the percentage of MDSC in tissue is correlated with the severity of intestinal inflammation [87, 88]. Sorted MDSC from murine colitis can suppress T cell proliferation in vitro, and adoptive transfer of MDSC sorted from murine colitis, generated in vitro, or exosome released by granulocytic MDSC can decrease intestinal inflammation and reduce the secretion of proinflammatory cytokines [87, 89].

Patients with inflammatory bowel disease (IBD) are at increased risk for developing colorectal cancer. It is been found that MDSC accumulation is further increased in the lesions during the progression from colitis to colorectal cancer [90]. Antibody-mediated depletion of MDSC in mice during colitis reduces colon tumor formation [91]. These results indicate that MDSC may play a role in the progression from colitis to colon cancer.

*(4) Macrophages and Natural Killer T (NKT) Cells*. Macrophages are white blood cells that reside in the tissues, which have critical roles in the host immune defenses [92]. Macrophages are differentiated from monocytes after emigrating from blood vessels in response to different stimuli [92, 93].

Intestinal macrophages are the most abundant mononuclear phagocytes in the intestine, especially in the large intestine, where they account for around one-fifth of all leucocytes [64, 94, 95]. Most of the macrophages are found underneath the epithelium of lamina propria of the intestine where they surveil the environment, phagocytose potential harmful antigens, and promote epithelial cell renewal by producing several mediators [95, 96], and some macrophages can also extend transepithelial dendrites into the intestinal lumen [95]. *α*4*β*7 integrin is important for homing of nonclassic monocyte to the gut, and impaired *α*4*β*7-dependent gut homing is associated with reduced and delayed wound healing and reduces perilesional presence of wound healing macrophages [97]. Intestinal macrophages play critical roles in maintaining intestinal homeostasis and are also drivers of the pathology associated with IBD [65, 94]. Resident macrophages in the lamina propria immediately capture and clear the bacteria that breach the epithelial layer without initiating an inflammatory response and thus are vital for maintaining homeostasis [94]. For instance, they efficiently eradicate phagocytosed enteric bacteria such as *Salmonella typhimurium* and *Escherichia coli*. They might also eliminate apoptotic and senescent cells and other cellular debris [64, 94]. Moreover, resident macrophages in the lamina propria have a unique surface marker's expression pattern-high expression of CX3CR1 and low expression of costimulatory molecules, Fc receptors for IgA and IgG, complement receptors, and integrin *α*2*β*1 [64, 98]. It suggests that these macrophages do not function as professional APC, unlike macrophages from other body compartments [64, 93, 99, 100]. These macrophages do not secret proinflammatory cytokines in reaction to cytokines or PAMPs or following phagocytosis of apoptotic cells [64, 93].

On the other hand, many inhibitory receptors are expressed on intestinal macrophages, including CD172a, CD200R1, IL-10R, and TGF-*β* receptors [94]. So the function of intestinal macrophages is influenced by corresponding soluble factors, such as IL-10 and TGF-*β*, secreted by a wide range of cell types, including epithelial cells, fibroblasts, subepithelial myofibroblasts, and lymphocytes [64]. Macrophages also have roles in tolerance through inducing anergic T cells or Treg and can impact the differentiation of naïve T cells into Th1, Th2, or Th17 cell types [101].

Studies have indicated the role of macrophages in the pathogenesis of IBD. In IBD patients, the number of macrophages increases in the inflamed mucosa with the major CD14^hi^HLA-DR^dim^ macrophages which can initiate a rapid response to luminal microbial antigens, unlike the resident macrophages [64, 102]. Also, the phenotype and functions of the macrophages in the inflamed sites differ from those in physical conditions. For instance, they express high levels of costimulatory molecules, such as CD40, CD86, CD80, and CD40 [103]. The CD14^hi^ macrophages in inflamed Crohn's ileum exclusively contain CD163^lo^CD11c^hi^ subset, while CD14^hi^ macrophages from noninflamed colon tissue contain both CD163^lo^CD11c^hi^ and CD163^hi^CD11c^lo^ subsets [98]. In addition, aberrant CD14-expressing macrophages isolated from the mucosa of IBD patients produce high levels of IL-12 and IL-23 in vitro under the microbial stimulation [104].

Animal models of IBD also support the role of dysregulated macrophages in the pathogenesis of IBD [64]. Murine colitis models show increased infiltration of CCL2 or MCP-1-mediated recruitment of monocytes and immature macrophages into the gut mucosa, which are arrested for further differentiation during inflammation, and they produce a large amount of proinflammatory mediators, such as TNF, IL-6, and nitric oxide [96]. IL-10^−/−^ mice spontaneously develop colitis in which macrophages preferentially differentiated into proinflammatory subsets that produce high levels of IL-12 and IL-23. Deficiency of macrophages in IL-10^−/−^ mice prevents the progression of colitis [105]. These results demonstrate that macrophages favor the development of intestinal inflammation.

NKT cell is another cell type involved in the pathogenesis of IBD [11]. NKT cell is a subset of lymphocytes that coexpress TCR along with typical surface receptors of natural killer cells and share the features of both innate and adaptive immune cells [106, 107]. NKT cell recognizes phospholipids or glycolipids that are presented by CD1d on the APC resulting in a rapid innate response through producing large amounts of Th1, Th2, and Th17 cytokines that then initiate most branches of the innate and adaptive immune systems [11]. NKT cell can be activated through multiple mechanisms, including direct activation by the recognition of CD1d on self- or microbial-derived lipids and indirect activation via cytokines, such as IL-12 and IL-18 [106]. Increased numbers of T cells expressing the NK marker CD161 are found in the inflamed lamina propria of ulcerative colitis patients, not in Crohn's disease. These cells can respond to CD1d with increased production of IL-13 [108]. In consistent with this, deficiency of CD1d and NKT cell prevents the development of oxazolone-induced murine colitis, resembling ulcerative colitis [109].

*(5) Innate Immune Cytokine Pathways*. In IBD, there is a markedly increased local production of various nonspecific inflammatory mediators, such as free radicals, leukotrienes, chemokines, and proinflammatory cytokines (e.g., TNF and TNF-related cytokines and IL-6 family of cytokines: IL-12, IL-23, IL-17, IL-18, and TGF-*β*) which follow the influx of inflammatory cells into the intestinal tissue [10]. Targeting those proinflammatory cytokines via monoclonal antibody or peptide-based virus-like particle vaccine strategy has been tested to be effective in the treatment of murine colitis and/or IBD patients [110–120].

*(6) TNF and TNF-Related Cytokines (TL1A)*. TNF is a 17 kDa proinflammatory cytokine mainly secreted by monocytes, macrophages, and T cells that can impact proliferation, differentiation, and functions of multiple types of cells [121]. TNF has multiple biological functions, including stimulation of the acute phase response, cachexia, cytotoxicity, and potentially lethal shock [121]. TNF can also promote the production of IL-1 and IL-6, enhance the expression of adhesion molecules, and stimulate fibroblast proliferation [121]. TNF exists as a transmembrane protein, named membrane-bound TNF, where it is cleaved to a soluble form by TNF-converting enzyme [122]. Secreted TNF employs its biological functions via binding to two distinct cell surface receptors, the 55 kDa TNFR1 (p55) and the 75 kDa TNFR2 (p75) [122]. The binding of TNF to its receptors leads to activation of one of the three pathways: a death domain pathway results in apoptosis; another activates JNK, which is involved in cell differentiation and proliferation; and the third pathway activates NF-*κ*B [123].

TNF has been implicated as an inflammatory mediator in many autoimmune diseases, such as rheumatoid arthritis, IBD, and multiple sclerosis [124]. Evidence has shown that the levels of TNF are increased in the intestinal mucosa, stool, and blood samples of IBD patients [123]. Moreover, the levels of TNF are correlated with clinical disease activity of Crohn's disease patients [123]. Several animal colitis models also demonstrate the role of TNF in the pathogenesis of intestinal inflammation. Anti-TNF monoclonal antibodies induce beneficial responses in some patients with IBD [123]. Anti-TNF blockade can not only promote the apoptosis of activated T cells but can also protect epithelial cells from apoptosis and tight junction compromise in the gastrointestinal epithelium [123].

More recently, TNF-like ligand 1A (TL1A) has been shown to be an important mediator of intestinal inflammation [125]. TL1A secretion is induced in APC by TLR ligands and FcR cross-linking, in CX_3_CR1^+^ mononuclear phagocytes by IBD-associated adherent microbiota and in T cells by TCR stimulation [126, 127]. The signaling pathway of TL1A is mediated through DR3, a TNF-family receptor that is mainly expressed on T cells [126]. TL1A synergistically increases the capacities of IL-12, IL-4, or IL-23 in the differentiation of Th1, Th2, and Th17 cells [125]. For instance, DR3 is selectively increased on Th17 cells, and TL1A enhances the proliferation of Th17 effector cells, whiles DCs derived from TL1A-deficient mice show a reduced capacity in promoting Th17 differentiation and proliferation [128].

The role of TL1A in the pathogenesis of IBD has been indicated [126]. The levels of TL1A are increased in IBD patients. Lamina propria CD14^+^ macrophages in Crohn's disease patients produce a higher level of TL1A, and TL1A promotes alloantigen-induced IL-17 and IFN-*γ* production from T cells [129]. Furthermore, it has been demonstrated that polymorphisms in the TL1A gene (TNFSF15) are associated with increased risk for IBD [130]. Reducing the expression of TL1A/TNFSF15 on monocytes and macrophage is associated with susceptibility to IBD [131].

Consistent with studies of TL1A in IBD patients, animal studies also demonstrate a role for TL1A [132, 133]. TL1A promotes group 3 innate lymphoid cells to produce IL-22 which can protect acute colitis by promoting mucosal healing [127]; furthermore, TL1A induces OX40L expression on group 3 innate lymphoid cells which stimulates T cell activation and is required for T cell-driven murine colitis [127]. TL1A can impair the intestinal epithelial barrier and regulate tight junction protein expression via several pathways in DSS colitis [134]. TL1A may promote the differentiation of Th9 cells and enhance IL-9 secretion by upregulating the expression of TGF-*β*, IL-4, and PU.1, thus exacerbating DSS-induced murine colitis [135]. Administration of exogenous TL1A to mice with DSS-induced colitis upregulates both Th1 and Th17 responses in inflamed colonic tissue [129]. The expression level of TL1A affects the expansion and function of Treg in modulating murine colitis [136]. Administration of anti-TL1A antibodies partially ameliorates DSS-induced murine colitis, completely prevents the development of TNBS-induced murine colitis, and reduces the intestinal fibrosis in a chronic colitis model [132, 133, 137]. Taken together, TL1A is indicated in IBD pathogenesis, modulating the severity of intestinal inflammation and fibrosis.

*(7) IL-6*. There is accumulating evidence that IL-6 plays a pivotal role in the pathogenesis of IBD [122, 138]. Studies have shown that the levels of IL-6 are increased in the serum and the intestinal mucosa of patients with active Crohn's disease [139]. Moreover, the level of IL-6 is positively correlated with the clinical disease activity, frequency of relapses, and the severity of endoscopic and histopathological signs of inflammation in Crohn's disease [122, 140, 141]. Macrophages and T cells in lamina propria are likely to be the main producers of IL-6 [122, 142].

In intestinal inflammation, IL-6 exerts its effect through binding to the soluble form of its corresponding receptor (sIL-6R), not through the membrane-bound receptor for IL-6 (IL-6R) [122, 143]. The levels of sIL-6R and IL-6/sIL-6R complex are increased in the serum of IBD patients. Then the IL-6/sIL-6R complex activates gp130-positive T cells lacking IL-6R, leading to the translocation of STAT-3 and subsequent activation of transcription of the antiapoptotic genes Bcl-2 and Bcl-xl [144]. Therefore, this pathway confers resistance against apoptosis of intestinal T cells in IBD patients and in animal models of colitis as well [122]. A clinical trial shows that tocilizumab, a humanized anti-IL-6R monoclonal antibody, induces significantly higher clinical response rate in active Crohn's disease than that of the placebo group [145]. It indicates that anti-IL-6R antibody may represent another therapeutic strategy for the management of IBD [122, 138].

#### 2.4.2. Adaptive Immune System Dysregulation

Dysregulation of the innate immune system causes functional abnormalities of the adaptive immune system, which reveals many characteristics of chronic inflammatory processes in IBD [64].

CD4^+^ Th cells play a critical role in orchestrating adaptive immune responses to various infections microbes [146, 147]. They are also involved in the pathogenesis of autoimmune and allergic diseases. Upon activation by T-cell receptor complex, naïve CD4^+^ T cells may differentiate into different types of Th cells in the presence of different cytokines, including Th1, Th2, Th17, and Th9, and inducible regulatory T (iTreg) cells. They can be characterized by their special cytokine production profiles, transcription factors, and their functions [148]. Under the stimulation of IL-12, naïve CD4^+^ T cells differentiate into Th1 cells, mainly producing IFN-*γ* and vital for protective immunity against intracellular viral and bacterial infections [148]. Under the stimulation of IL-4, naïve CD4^+^ T cells differentiate into Th2 cells, producing IL-4, IL-5, IL-13, and IL-25, and are critical for eliminating extracellular parasites such as helminths [148]. In the presence of IL-4 and TGF-*β*, naïve CD4^+^ T cells may differentiate into Th9 cells secreting IL-9, IL-10, and IL-21, which regulate allergic inflammation, autoimmune inflammation, and antitumor immunity [149]. TGF-*β* and IL-6 induce naïve CD4^+^ T cells to differentiate into Th17 cells, producing IL-17, IL-17F, IL-21, and IL-22, important for controlling extracellular bacterial and fungi infections [148]. In the presence of TGF-*β* without IL-6, naïve CD4^+^ T cells differentiate into iTreg cells. iTreg cells together with naturally occurring regulatory T (nTreg) cells are vital for the maintenance of immune tolerance and regulation of lymphocyte homeostasis, activation, and function [148]. Transcription factors also play important roles in the differentiation of Th cells and production of cytokines. The vital transcription factors of Th lineage are T-bet/Stat4 for Th1, GATA-3/Stat5 for Th2, PU.1/Smad/Stat6 for Th9, ROR*γ*t/Stat3 for Th17, and Foxp3/Stat5 for iTreg [148, 149].

It has been widely accepted that Crohn's disease is caused by an overly aggressive Th1 immune response and recently found excessive IL-23/Th17 pathway activation to bacterial antigens in genetically predisposed individuals [10, 52, 150–152]. The resulting infiltration of the bowel by granulocytes and macrophages leads to a release of enzymes, reactive oxygen intermediates, and proinflammatory cytokines, all of which cause discontinuous ulceration and full thickness bowel wall inflammation often including granulomas [153, 154]. On the contrary, ulcerative colitis is usually considered a “Th2-like” disease characterized by increased amounts of IL-5 and IL-13 [126]. Furthermore, Th17 and Treg are implicated in both Crohn's disease and ulcerative colitis, while Th9 cells are predominately involved in the pathogenesis of ulcerative colitis.

*(1) Th1 Cells*. A number of observations indicate Th1 cells are involved in the pathogenesis of Crohn's disease [126, 155]. T cells in the colonic lamina propria of Crohn's disease patients produce large amounts of IFN-*γ* and increase the expression of IL-12R*β*2, T-bet, and STAT4 [155]. IFN-*γ*-producing lamina propria lymphocytes are accumulated in the mucosa of patients. Macrophages in Crohn's disease patients produce high levels of IL-12 [155]. At the initial phase of Crohn's disease, mucosal T cells mount a typical Th1 response that resembles an acute infectious process and gradually disappear with progression to late Crohn's disease [156]. In addition, clinical responses are induced in a subcohort of patients with Crohn's disease treated with anti-IFN-*γ* antibody [126, 155]. In an animal colitis model, abrogation of IFN-*γ* in the CD4^+^ CD45RB^hi^/Rag^−/−^ transfer model potently prevents the development of colitis; T-bet-deficient CD4^+^CD45RB^hi^ cell cannot induce the colitis in Rag^−/−^ recipients [157]. These results indicate that Th1 plays a role in the pathogenesis of Crohn's disease.

*(2) Th9 Cells*. The role of Th9 and IL-9 in the pathogenesis of ulcerative colitis has been identified recently [158, 159]. The percentages of PU.1^+^IL-9^+^Th9 cells are significantly increased in colonic lamina propria of patients with ulcerative colitis, especially in patients with active ulcerative colitis [158]; IL-9 mRNA expression is also increased in inflamed colon samples from patients with ulcerative colitis [159]. Consistent with increased IL-9, IL-9R is found overexpressed on gut epithelial cells [158, 159]. Adoptive transfer of Th9 cells results in exaggerating intestinal inflammation of RAG^−/−^ mice, while deficiency in PU.1 and IL-9 in T cells prevents oxazolone-induced murine colitis [158]. However, one recent study shows that the cytokine and colitis-inducing potential of Th9 is controlled by CD96 expression: adoptive transfer of CD96^low^ Th9 into Rag1^−/−^ mice induces severe intestinal inflammation, while transfer of CD96^high^ Th9 does not cause colitis and blockade of CD96 can restore the expansion and inflammatory properties of CD96^high^ Th9 cells [160], which indicates a functional heterogenicity of Th9 cells. Deficiency of IL-9 suppresses TNBS-induced murine colitis and reduces the number of PU.1^+^T cells in the lamina propria [161]. Further, it shows that IL-9 exacerbates murine intestinal inflammation through regulating intestinal tight junction, mucosa permeability, and mucosal wound healing [158, 161]. Administration of IL-9 blocking antibody improved oxazolone-induced murine colitis [158]. These results suggest the proinflammatory role of the Th9/IL-9 pathway in IBD, especially in ulcerative colitis.

*(3) Th17 Cells*. With the finding of the IL-23/Th17 pathway, more recently, studies highlight the role of this pathway in the pathogenesis of IBD [162]. Studies have shown that large amounts of IL-17-producing cells are mainly accumulated in the lamina propria of ulcerative colitis patients and in the submucosa and muscularis propria of Crohn's disease patients [163]. Flow cytometry analysis of mucosal cells also shows that the number of IL-17 producing T cells is increased in Crohn's disease patients than in normal controls, but some of these cells coexpress IFN-*γ* [164]. Gut biopsies grown ex vivo and LPMC cultured in vitro also produce high levels of IL-17 in IBD patients than in controls [165]. Other Th17 cytokines, such as IL-21, IL-22, and IL-23, are also increased in the inflamed tissue of IBD patients [166]. Also, GWAS suggest at least over 20 SNPs are linked to loci associated with Th17-regulating intracellular networks and signal transduction, indicating an important role of Th17 towards the pathogenesis of IBD, including IL-23R, IL-12B, JAK2, STAT3, and CCR6 [167].

The role of Th17 cells in the pathogenesis of IBD has also been evaluated in animal models. IL-17 is shown to be elevated in the IL-10 knockout and RAG1 knockout mouse models of IBD, respectively [168, 169]. Anti-IL-17 Ab ameliorates the severity of intestinal inflammation in RAG1 knockout mice reconstituted with IL-10 knockout CD4^+^ T cells [169]. Further, deficiency of IL-17R (receptor) prevents the development of TNBS-induced murine colitis, including improving body weight loss, decreasing productions of IL-6 and local macrophage inflammatory protein-2, ameliorating colonic inflammation, and reducing tissue myeloperoxidase activity [170]. IL-17F-deficiency improves the development of DSS-induced murine colitis, whereas IL-17-deficiency exaggerates the development of DSS-induced murine colitis, indicating that IL-17F rather than IL-17A is important in sustaining DSS colitis [171]. This has been shown to be important clinically: monoclonal antibody against IL-17A, secukinumab, is ineffective in treating Crohn's disease but causes a higher rate of adverse events and increases disease severity [48, 172].

Enhanced production of IL-17 in the gut is also found in the C3H/HeSnJ SCID transfer colitis model, and adoptive transfer of IL-17-producing T cells to SCID recipients leads to severe colitis [173]. In the model of CD8^+^ T cell-dependent colitis, it shows that a single adoptive transfer of naïve CD8^+^ T cells into syngeneic RAG-deficient mice results in severe colitis, with rapid spontaneous proliferation of these CD8^+^ T cells in MLN [174]. These CD8^+^ T cells in the MLN coexpress IL-17 and IFN-*γ*. Also, adoptive transfer of naïve CD8^+^ T cells isolated from either IL-17- or IFN-*γ*-deficient mice induced a remarkably less severe colitis, suggesting IL-17 and IFN-*γ* can cooperate to cause colitis in this model [174].

A role for IL-21 in the murine colitis is also indicated [175]. DSS colitis and TNBS-relapsing colitis are significantly decreased in IL-21-deficient mice, which is associated with reduced expression of Th17 cell-related genes (IL-17, IL-17F, and ROR*γ*t) in the colon tissue [175]. Furthermore, blockade of IL-21 using a specific IL-21R-fusion protein improves intestinal inflammation and downregulates Th17 responses during the course of DSS colitis [175]. Taken together, these data indicate that the Th17 pathway plays an important role in the pathogenesis of IBD.

*(4) Treg Cells*. The GALT is believed to be the primary site where naïve conventional CD4^+^ T cells convert to iTreg after exposure to oral antigens in a lymphogenic environment [176, 177]. This conversion is dependent on TGF-*β* and retinoic acid producing CD103^+^ DCs in the GALT. It has been supposed that nTreg mainly protects against autoimmunity in situ, but iTreg primarily inhibits immune responses against environmental and food antigens in the gut [178].

The dysfunction of Treg in IBD is usually believed to be due to the defective numbers of Treg or their suppressive function which cannot control the intestinal inflammation [177]. For instance, patients with a *FOXP3* gene mutation have defective Treg and always suffer from intestinal inflammation [179]. When compared with healthy controls, the numbers of Treg are decreased in peripheral blood but increased in inflamed colons of patients with IBD [177]. Also, the ratio of Treg to Th17 in peripheral blood is reduced in IBD patients when compared with controls [180]. However, the increased number of Treg in the colon lamina propria of IBD patients is still lower than that of patients with infectious enteritis or diverticulitis [181]. Treg isolated from inflamed colon or peripheral blood maintained normal cell contact-dependent, cytokine-independent suppressive capacity in vitro [181–183]. But effector T cells from IBD patients display relative resistance to Treg-mediated suppression, because effector T cells express high levels of Smad7 which is an inhibitor of the TGF-*β* signaling pathway [184]. These data indicate that Treg dysfunction might be due to an extrinsic milieu of activated cells that are resistant to suppression [177].

Studies on animal models of colitis also demonstrate the role of Treg in the control of intestinal inflammation [177]. Adoptive transfer of naïve T effector cells in the absence of Treg into SCID mice leads to colitis, whereas cotransfer of T effector cells and Treg does not induce colitis [177]. Furthermore, adoptive transfer of CD4^+^CD25^+^ Treg cures established CD4^+^CD45RB^hi^ transfer colitis [185]. In this model, Treg are capable of suppressing colonic inflammation by downregulating Th1 and Th17 responses depending on the presence of IL-10 and TGF-*β* [186, 187].

More recently, a new type of iTreg, called iTR35, has been identified which mainly produces the suppressive cytokine IL-35, not IL-10 or TGF-*β* [188, 189]. Adoptive transfer of IL-35-deficient Treg cannot cure CD4^+^CD45RB^hi^-induced murine colitis [189], whereas adoptive transfer of iTR35 generated in vitro can significantly improve the intestinal inflammation [188]. IL-35 also shows strong function in controlling intestinal inflammation. Administration of recombinant IL-35 significantly reduces the development of several forms of experimental colitis and reduces levels of cytokines of Th1 and Th17 cells [190].

Both iTreg and Th17 differentiations require TGF-*β* which induces Foxp3 and ROR*γ*t, so there is a fine balance existing between these two types of cells under the control of many factors [11]. For instance, low concentrations of TGF-*β* together with IL-6 and IL-21 induce the expression of IL-23R and promote the differentiation of Th17 cells [11]. On the contrary, high concentrations of TGF-*β* inhibit the expression of IL-23R and promote the development of iTreg [11]. Foxp3 directly interacts with ROR*γ*t to suppress its function, but IL-6, IL-21, and IL-23 downregulate the Foxp3-mediated suppression of ROR*γ*t [11, 191]. On the other hand, there is a close relationship between these two types of cells. Recent data have documented that memory Treg can convert into Th17 cells under inflammatory conditions, in which IL-1 is the key molecule in promoting conversion [192, 193]. A hybrid subpopulation of memory Treg coexpressing Foxp3 and ROR*γ*t has been found which exert suppressive functions but concomitantly secret IL-17 ex vivo [194–196]. In the presence of IL-1, IL-2, IL-23, and TGF-*β*, human Th17 cells preferentially differentiate from natural naïve regulatory cells, rather than from conventional CD4^+^CD25^−^ naïve T cells [197]. Together, these findings illustrate that Treg deficiency may be associated with the pathogenesis of IBD.

## 3. Summary

IBD is a chronic and life-threating disease characterized by episodes of intestinal inflammation. Substantial progress in the past several decades has greatly increased our understanding of the pathophysiology of IBD especially in the field of immunology and increased the opportunities to explore other therapeutic pathways/targets. However, there are still unknown questions on pathogenesis, disease behavior, and intestinal inflammation drivers in different IBD patient subgroups which require further exploration. Associations between proinflammatory and anti-inflammatory immune cells and molecules and various genetic susceptibility, intestinal microbiota, and environmental factors (e.g., diet, smoking, and physiological stress) are continuously being evaluated to allow for a comprehensive understanding of pathogenesis of IBD. These investigations are critical not only for developing novel treatment strategies including the selection of the right targets to optimally manage IBD such as fecal microbiota transplantation, antisense oligonucleotide targeting proinflammatory molecules (such as NF-*κ*B and Intercellular adhesion molecule 1), and monoclonal antibody/biologics to neutralize proinflammatory cytokines (such as TNF and IL-12/IL-23p40) but also for identifying biomarkers for diagnosis, monitoring, and prognostics or prediction of disease progress and treatment outcome. With this knowledge, we may have the ability to develop novel personalized treatments for IBD patients.

## Abbreviations

Ab: Antibody
APC: Antigen-presenting cells
DC: Dendritic cells
DSS: Dextran sodium sulfate
IBD: Inflammatory bowel disease
IEC: Intestinal epithelial cells
IL: Interleukin
GALT: Gut-associated lymphoid tissue
GWAS: Genome-wide association studies
MDSC: Myeloid-derived suppressor cells
MLN: Mesenteric lymph nodes
NOD2: Nucleotide-binding oligomerization domain 2
NLR: Nod-like receptor
NF-*κ*B: Nuclear factor kappa B
NKT: Natural killer T
PAMPs: Pathogen-associated molecular patterns
PRRs: Pattern recognition receptors
SNP: Single-nucleotide polymorphisms
TGF-*β*: Transforming growth factor beta
Th: T helper cells
Treg: Regulatory T cell
TLR: Toll-like receptor
TL1A: TNF-like ligand 1A
TNBS: Trinitrobenzenesulfonic acid
TSLP: Thymic stromal lymphopoietin.
